# Prevalence of Actionable Exposures to Pharmacogenetic Medications Among Solid Organ Transplant Recipients in a Population-Scale Biobank

**DOI:** 10.3390/jpm15050185

**Published:** 2025-05-02

**Authors:** Alaa Radwan, Kimberly M. Deininger, Amrut V. Ambardekar, Heather D. Anderson, Nicholas Rafaels, Laura M. Saba, The Colorado Center for Personalized Medicine, Christina L. Aquilante

**Affiliations:** 1 Department of Pharmaceutical Sciences, University of Colorado Skaggs School of Pharmacy and Pharmaceutical Sciences, Aurora, CO 80045, USA; alaa.radwan@cuanschutz.edu (A.R.); kimberly.deininger@cuanschutz.edu (K.M.D.); laura.saba@cuanschutz.edu (L.M.S.); 2 Colorado Center for Personalized Medicine, University of Colorado Anschutz Medical Campus, Aurora, CO 80045, USA; heather.anderson@cuanschutz.edu (H.D.A.); nicholas.rafaels@cuanschutz.edu (N.R.); ccpm-biobank@cuanschutz.edu (CCPM); 3 Division of Cardiology, Department of Medicine, University of Colorado School of Medicine, Aurora, CO 80045, USA; amrut.ambardekar@cuanschutz.edu; 4 Department of Clinical Pharmacy, University of Colorado Skaggs School of Pharmacy and Pharmaceutical Sciences, Aurora, CO 80045, USA

**Keywords:** pharmacogenetics, pharmacogenomics, solid organ transplant, biobank

## Abstract

**Background/Objectives**: Solid organ transplant (SOT) recipients are exposed to multiple medications, many of which have pharmacogenetic (PGx) prescribing recommendations. This study leveraged data from a population-scale biobank and an enterprise data warehouse to determine the prevalence of actionable exposures to PGx medications among kidney, heart, and lung transplant recipients during the first six months post-transplant. **Methods**: We conducted a retrospective analysis of adult SOT patients with genetic data available from the Colorado Center for Personalized Medicine (CCPM) biobank and clinical data from Health Data Compass (HDC). We evaluated 29 variants in 13 pharmacogenes and 42 Clinical Pharmacogenetics Implementation Consortium (CPIC) level A or B medications (i.e., sufficient evidence to recommend at least one prescribing action based on genetics). The primary outcome was actionable exposure to a PGx medication (i.e., actionable phenotype and a prescription for an affected PGx medication). **Results**: The study included 358 patients. All patients were prescribed at least one PGx medication, and 49.4% had at least one actionable exposure to a PGx medication during the first six months post-transplant. The frequency of actionable exposure was highest for tacrolimus (15.4%), followed by proton pump inhibitors (PPIs) (15.1%) and statins (12.8%). Statin actionable exposures significantly differed by transplant type, likely due to variations in prescribing patterns and actionable phenotypes for individual statins. **Conclusions**: Our findings highlight the potential clinical utility of PGx testing among SOT patients. Further studies are needed to address the impact on clinical outcomes and the optimal timing of PGx testing in the SOT population.

## 1. Introduction

Solid organ transplant (SOT) is the treatment of choice for end-stage organ failure [1]. The number of SOTs performed each year has steadily increased over the last decade, with over 45,000 SOTs performed in the United States in 2023 [2]. Immunosuppressants are the cornerstone of SOT pharmacotherapy and consist of a cocktail of medications that have substantially improved SOT clinical care. As a result, one-year allograft and patient survival rates now approach 90% [3]. Long-term survival after SOT has also improved; median survival following SOT ranges from about 6 years after lung transplantation to 27 years after kidney transplantation [4,5]. Despite improvements in survival rates, SOT recipients experience numerous comorbidities (e.g., hyperlipidemia, gastrointestinal issues, cardiovascular disease) over time due to increasing age, prolonged administration of immunosuppressants and their associated side effects, and other patient-specific risk factors [6]. Therefore, SOT recipients often have a high medication burden, receiving an average of 10 to 14 medications per day; thus, it is critical to maximize drug efficacy and minimize toxicities in this population [6,7,8].

Pharmacogenetics (PGx) is a component of precision medicine that studies how genetic variation contributes to interindividual variability in drug disposition, response, and toxicity, with the goal of using genetic results to inform medication prescribing [9]. The Clinical Pharmacogenetics Implementation Consortium (CPIC) publishes evidence-based clinical practice guidelines to facilitate the use of PGx test results in clinical practice. Currently, there are 28 CPIC guidelines covering 164 medications and 34 genes [10]. Many of these medications are commonly used in SOT recipients. For example, tacrolimus and azathioprine are immunosuppressants used to prevent allograft rejection, with CPIC guidelines recommending dose modifications in individuals carrying certain variants in *CYP3A5*, *TPMT*, and *NUDT15* genes [11,12]. Many non-immunosuppressant medications used in SOT also have CPIC guidelines, including statins, proton pump inhibitors (PPIs), antiemetics, warfarin, clopidogrel, voriconazole, opioids, and antidepressants, among others [13,14,15,16,17,18,19,20,21].

Variants in PGx genes are highly prevalent in the general population, and SOT recipients are no exception [22,23,24]. In a recent kidney transplant PGx study, all patients had at least one actionable PGx variant [23] and were prescribed at least one medication with an actionable PGx biomarker [25]. In addition, a small study of kidney transplant recipients of European and Native American descent found that more than one-third of patients undergoing prospective PGx testing had a potentially actionable drug-gene interaction [26]. However, several gaps in knowledge remain regarding the potential clinical utility of PGx testing in the SOT population, particularly in large cohorts and different types of SOT (e.g., heart, lung). Therefore, the objective of our study was to use patient-level genetic and medication data to determine the prevalence of actionable exposures to PGx medications among SOT recipients during the first six months post-transplant. Unique to the SOT PGx literature, we leveraged the biobank at the Colorado Center for Personalized Medicine (CCPM biobank), a population-scale, dual-purpose research and clinical resource, and Health Data Compass (HDC), an enterprise data warehouse, to conduct this investigation [27,28].

## 2. Methods

### 2.1. Data Sources and Study Population

We conducted a retrospective cohort study using patient data obtained from the electronic health record (EHR) at UCHealth in Aurora, Colorado, USA. Data are stored and managed by HDC, an enterprise data warehouse (https://www.healthdatacompass.org, accessed on 11 March 2025). All patients who are seen at UCHealth for any reason and have an EHR in the UCHealth system are included in the HDC data warehouse. The data warehouse also includes genetic data for patients who have participated in the CCPM biobank research study [28]. Briefly, the CCPM biobank collects, stores, and generates genetic data on participants’ blood samples, with the goal of advancing personalized medicine research and clinical care. UCHealth patients 18 years of age and older may enroll in the CCPM biobank via an electronic self-consent model through their UCHealth online patient portal, My Health Connection (Epic Systems Corporation, Verona, WI, USA). A unified consent form approach has been used since 2019, which authorizes consent for research, participant recontact, use in industry partnerships, and return of clinical genetic test results to the EHR.

For this investigation, we included adult kidney, heart, and lung transplant recipients who had their transplant performed at UCHealth between 1 February 2011 and 9 August 2020. To be included, patients needed to have demographic, clinical, medication, and genetic data available in the HDC data warehouse and be enrolled in the CCPM biobank. They also needed to be at least six months post-transplant. We excluded patients with multiple organ transplants (e.g., kidney/pancreas) and those who were retransplanted within 6 months of the initial transplant date. We excluded liver transplant recipients to avoid issues arising from discordant donor and recipient genotypes, as the CCPM biobank does not include donor liver genetic information. The Colorado Multiple Institutional Review Board approved our study as secondary use of clinical data with a HIPAA waiver of consent.

### 2.2. Variant Genotyping and Imputation

Patient blood samples were genotyped as part of the CCPM biobank research study using a customized version of the Illumina Infinium Expanded Multiethnic Global Array (MEGA^EX^), which included over two million variants and was enriched to cover over 17,000 PGx variants [27,28,29]. We used PLINK v1.9 to perform comprehensive quality control on samples and variants as previously described [30]. Variants that were not directly genotyped were imputed using the Michigan Imputation Server and the Haplotype Reference Consortium version r1.1 2016 as a reference panel [31].

We evaluated 29 variants in 13 PGx genes: *ABCG2*, *CYP2B6*, *CYP2C9*, *CYP2C19*, *CYP2C* cluster, *CYP3A5*, *CYP4F2*, *DPYD*, *SLCO1B1*, *NUDT15*, *TPMT*, *UGT1A1*, and *VKORC1. CYP2D6* was not evaluated since several common clinically relevant variants in this gene were not interrogated by MEGA^EX^. We created translation tables to assign diplotypes and phenotypes from variant data. We used PharmGKB allele definition and diplotype-phenotype tables as the reference for star allele, diplotype, and phenotype assignments [32]. *VKORC1* and *CYP4F2* phenotypes were assigned as previously described in the literature [23].

### 2.3. PGx Medications and Actionable Exposures

We evaluated 42 CPIC level A or B medications available in the United States and affected by variants in the 13 evaluated genes (Supplemental Table S1). We defined exposure to a PGx medication as at least one prescription for the drug between the date of transplant and six months post-transplant. The primary outcome of the study was actionable exposure to a PGx medication during the first six months post-transplant in SOT recipients transplanted between 1 February 2011 and 9 August 2020. Additionally, we compared the prevalence of PGx medication prescriptions and actionable exposures to PGx medications across different organ types during this period. Actionable exposure was defined as a patient possessing a PGx phenotype for which CPIC recommended a specific prescribing action, coupled with the patient receiving a prescription for the corresponding medication. First, we included PGx medications with CPIC recommendations of any strength (i.e., strong, moderate, or optional) and then performed a secondary analysis of PGx medications with only strong or moderate recommendations [33]. The prevalence of actionable exposure was calculated by dividing the number of patients who had at least one actionable exposure during the six months following their transplant by the total number of patients in our cohort. We compared the prevalence of medication prescriptions and actionable exposures during the first six months post-transplant across organ types using chi-square tests or Fisher’s exact tests. A *p*-value < 0.05 was considered statistically significant. Descriptive statistics and statistical comparisons were generated using R versions 3.6.0 and 4.4.0.

## 3. Results

### 3.1. Patient Characteristics and Demographics

The study included 358 SOT recipients, with 58.1% men, 81.3% self-reported European ancestry, and a mean (±SD) age at transplant of 52.7 ± 13.2 years. The cohort consisted of 72.1% kidney, 17.3% lung, and 10.6% heart transplant recipients. Patient characteristics are summarized in Table 1.

### 3.2. Diplotypes and Phenotypes

**Figure 1:**
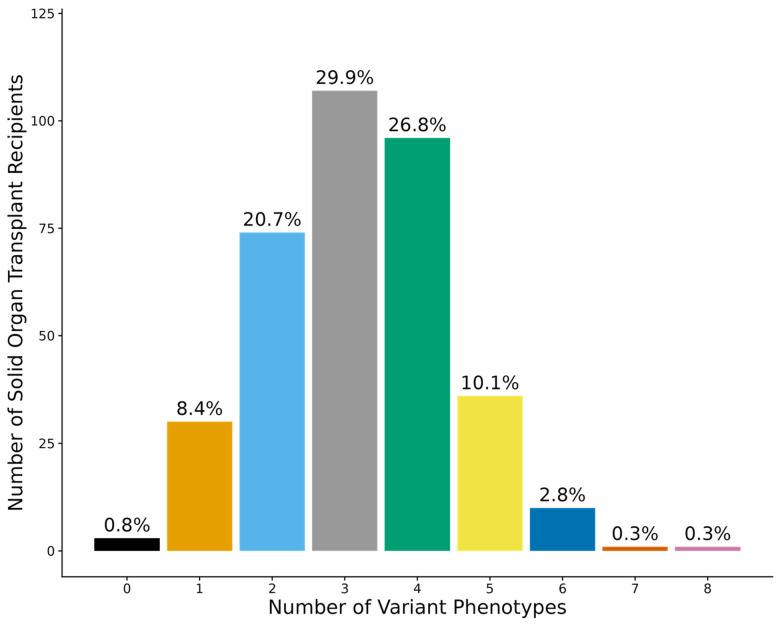
Number and percentage of solid organ transplant recipients in the Colorado Center for Personalized Medicine biobank (N = 358) with variant pharmacogenetic phenotypes.

All 29 variants were in Hardy-Weinberg equilibrium. The frequencies of PGx diplotypes and phenotypes are summarized in Table 2. Most patients (99.2%) had at least one variant PGx phenotype (Figure 1). Over two-thirds of patients (70.1%) had three or more variant PGx phenotypes (Figure 1).

### 3.3. PGx Medication Characteristics

**Figure 2:**
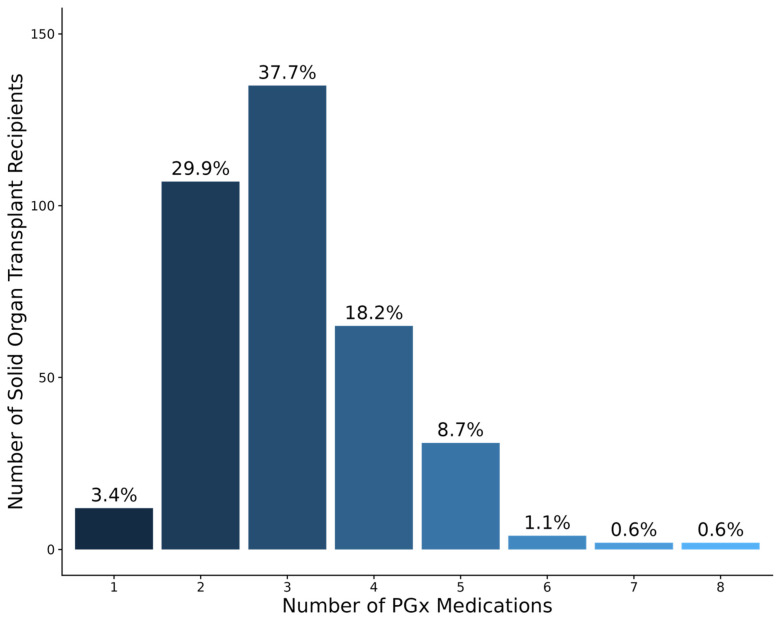
Number and percentage of solid organ transplant recipients in the Colorado Center for Personalized Medicine biobank (N = 358) prescribed pharmacogenetic medications during the first 6 months post-transplant.

Of the 42 CPIC level A and B medications evaluated, 21 (50%) were prescribed at least once in the cohort. All patients had at least one prescription for a PGx medication during the first 6 months post-transplant (Figure 2). The mean number of PGx medications prescribed per patient was 3.1 ± 1.1 (median = 3, range = 1–8). The percentage of patients prescribed ≥3, ≥4, and ≥5 PGx medications was 66.7%, 29.0%, and 10.8%, respectively (Figure 2).

### 3.4. Actionable Exposures to PGx Medications with Strong, Moderate, or Optional Prescribing Recommendations

**Figure 3:**
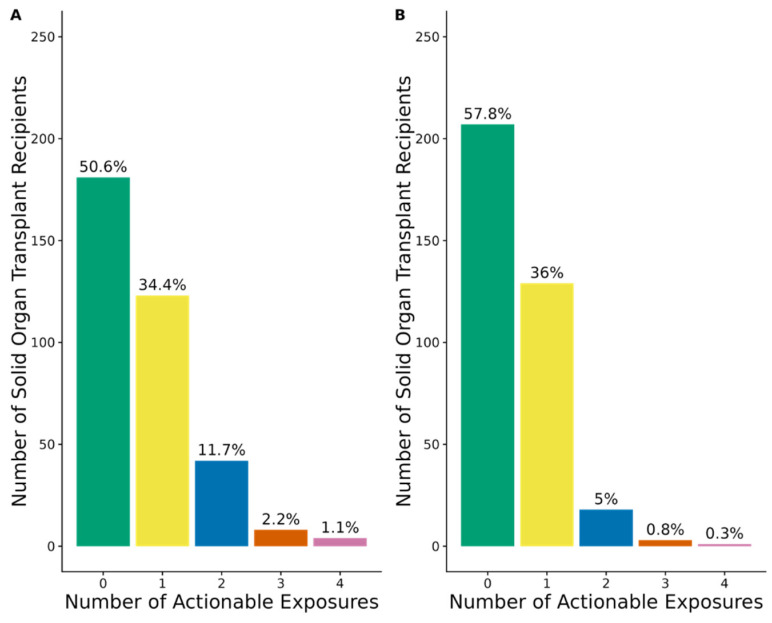
Among the 358 solid organ transplant recipients in the Colorado Center for Personalized Medicine biobank: (**A**) prevalence of actionable exposures to pharmacogenetic (PGx) medications with strong, moderate, or optional Clinical Pharmacogenetics Implementation Consortium (CPIC) guideline recommendations during the first six months post-transplant; (**B**) prevalence of actionable exposures to PGx medications with strong or moderate CPIC guideline recommendations during the first six months post-transplant.

In the entire SOT cohort (N = 358), 177 patients (49.4%) had at least one actionable exposure to a PGx medication with strong, moderate, or optional CPIC prescribing recommendations (Figure 3A). Of patients with at least one actionable exposure, the percentage of patients by transplant type was 69.5% kidney, 19.8% lung, and 10.7% heart. The percentage of patients with an actionable exposure did not differ significantly across the type of transplant: 47.7% for kidney recipients, 56.5% for lung recipients, and 50% for heart recipients (*p* = 0.46).

Table 3 outlines prescribing and actionable exposure frequencies by PGx medication and drug class in the entire cohort. The most frequently prescribed drug class was PPIs (*N* = 342, 95.5%), with 54 patients (15.8%) having an actionable exposure. PPI prescribing and actionable exposure frequencies did not differ by type of transplant (Supplemental Table S2). Tacrolimus was the most frequently prescribed transplant-specific PGx medication (*N* = 310, 86.6%), with an actionable exposure frequency of 17.7% in the overall cohort. The prescribing frequency of tacrolimus was highest in kidney (93.4%), followed by heart (92.1%) and lung (54.8%), *p* < 0.001. Tacrolimus actionable exposure frequency among kidney, heart, and lung transplant recipients was 17%, 25.7%, and 14.7%, respectively (*p* = 0.40). Thiopurines were less commonly prescribed (*N* = 65, 18.2%) in the overall cohort and had an actionable exposure frequency of 13.8%, which was primarily due to TPMT intermediate metabolizers. The prescribing frequency of azathioprine was highest in lung (87.1%), followed by kidney (4.3%) and heart (0%), *p* < 0.001. Azathioprine actionable exposure frequency among kidney and lung transplant recipients was 18.2% and 13.0%, respectively (*p* = 0.64).

In terms of non-transplant medications, statins were prescribed to over half (59.8%) of the patients in the cohort, with an actionable exposure frequency of 21.5%. The prescribing frequency of statins was highest in heart (100%), followed by kidney (57.0%) and lung (46.8%), *p* < 0.001. Statin actionable exposure frequency among kidney, heart, and lung transplant recipients was 27.2%, 5.3%, and 13.8%, respectively (*p* = 0.005). In contrast, warfarin, selective serotonin reuptake inhibitors (SSRIs), and voriconazole prescribing frequencies were lower in the overall cohort (13.1%, 11.5%, and 4.7%, respectively), but their actionable exposure frequencies were among the highest of all drugs (89.4%, 53.7%, and 47.1%, respectively). The prescribing frequency of warfarin was highest in lung (22.6%), followed by heart (13.2%) and kidney (10.9%), *p* = 0.049. Warfarin actionable exposure frequencies among kidney, heart, and lung transplant recipients were 82.1%, 100%, and 100%, respectively (*p* = 0.20). The prescribing frequency of SSRIs was highest in lung (19.4%), followed by heart (15.8%) and kidney (8.9%), *p* = 0.046. SSRI actionable exposure frequency among kidney, heart, and lung transplant recipients was 47.8%, 83.3%, and 50%, respectively (*p* = 0.34). The prescribing frequency of voriconazole was highest in lung (17.7%), followed by heart (7.9%) and kidney (1.2%), *p* < 0.001. Voriconazole actionable exposure frequency among kidney, heart, and lung transplant recipients was 0%, 100%, and 45.5%, respectively (*p* = 0.047). Other medications prescribed to less than 2% of the population are shown in Table 3.

### 3.5. Actionable Exposures to PGx Medications with Strong or Moderate Prescribing Recommendations

When the analysis was limited to PGx medications with strong or moderate CPIC prescribing recommendations (i.e., excluding PGx medications with optional recommendations), 151 patients (42.2%) in the entire SOT cohort had at least one actionable exposure (Figure 3B). Of those with at least one actionable exposure, the breakdown by transplant type was 70.2% kidney, 17.9% lung, and 11.9% heart transplant recipients. Within each transplant type, the percentage of patients with an actionable exposure was 106 of 258 kidney recipients (41.1%), 27 of 62 lung recipients (43.5%), and 18 of 38 heart recipients (47.4%), *p* = 0.74.

## 4. Discussion

In this study, we leveraged data from CCPM, a population-scale biobank, and HDC, an enterprise data warehouse, to evaluate the prevalence of actionable exposures to PGx medications in SOT recipients during the first six months following transplantation. Our approach highlights the feasibility and utility of biobanks and large data warehouses to answer clinical PGx questions, particularly in smaller patient populations, such as SOT. We found that nearly half of SOT recipients had at least one actionable exposure to a PGx medication. Lung recipients had the highest proportion of actionable exposures, followed by heart and kidney, although the difference was not statistically significant. When we evaluated each medication individually by transplant type, we observed significant differences in actionable exposures for some medications, such as statins and voriconazole.

We found that almost half of SOT recipients in our cohort had at least one actionable exposure to a PGx medication during the first six months post-transplant, suggesting that this population would likely derive benefit from PGx testing. Previously, Brady et al. reported an actionable exposure frequency of 36% in a small cohort of kidney transplant recipients (*N* = 81) [26]. Our finding of a higher actionable exposure frequency is likely due to several factors, including differences in organ transplant types between the two studies (i.e., kidney, heart, and lung vs. kidney only), time period of medication evaluation (first six months post-SOT vs. at the time PGx results were returned), and ancestry differences (i.e., more patients of European descent vs. more patients of Indigenous American descent). Together, these factors likely influenced the frequencies of prescribed medications, variant alleles, and actionable exposures between the two cohorts [26].

Consistent with previous findings in both transplant and non-transplant populations, we found that 99.2% of SOT recipients had at least one variant phenotype based on 29 variants in 13 genes. Nguyen et al. evaluated 12 pharmacogenes in kidney transplant recipients and found that 100% of patients had at least one variant non-normal phenotype [23]. Similar findings have been reported in large non-transplant biobank cohorts [22,24]. In terms of PGx medication prescribing patterns, all patients in our cohort were prescribed at least one PGx medication, with over two-thirds requiring three or more PGx medications in the first six months post-transplant. Our findings align with those from 490 kidney transplant recipients in the Taiwan Precision Medicine Initiative, where all patients had at least one PGx medication prescription [25]. These data collectively underscore the high prevalence of PGx variants and the significant PGx medication burden observed in transplant recipients, suggesting that SOT populations would be a worthwhile focus for PGx implementation initiatives.

Immunosuppressants, such as tacrolimus, are essential in managing SOT patients. In our cohort, tacrolimus was the most frequently prescribed transplant medication. Among those treated with tacrolimus, 17.7% had an actionable exposure (i.e., CYP3A5 expressors), indicating that a higher starting dose would be needed to attain target tacrolimus trough levels, per CPIC guidelines [11]. Therapeutic drug monitoring of tacrolimus trough concentrations is the most widely used strategy to individualize tacrolimus doses [34]. Although *CYP3A5*-guided dosing is associated with faster attainment of therapeutic drug concentrations, the impact of *CYP3A5* genotype-guided dosing on clinical outcomes remains inconsistent [11,35]. Besides CPIC, the International Association of Therapeutic Drug Monitoring and Clinical Toxicology issued a consensus report acknowledging the role of *CYP3A5* in initial tacrolimus dosing. The report suggests future studies to investigate population pharmacokinetic models that integrate *CYP3A* variants (and possibly other genetic data) to optimize tacrolimus therapy [35]. Azathioprine was prescribed mainly to lung transplant recipients, where 13% had an actionable exposure and would have required a lower starting dose to reduce the risk of myelosuppression [12]. In addition to immunosuppressants, the highest frequencies of actionable exposures were observed for PPIs (15.1%), statins (12.8%), and warfarin (11.7%). Although warfarin prescribing frequency was relatively low in our cohort, its actionable exposure frequency was among the highest; over 55% of patients in this cohort were carriers of the *VKORC1 rs9923231* variant allele, which is associated with altered warfarin sensitivity. Similarly, SSRIs and voriconazole actionable exposure frequencies were relatively high due to common polymorphic CYP2C19 phenotypes (i.e., rapid and intermediate metabolizers). Our prescribing frequency and actionable exposure data suggest that preemptive multigene panel testing, with genes that impact both immunosuppressant and non-immunosuppressant medications, may be advantageous in the SOT setting [26,36]. Some institutions have begun to implement this approach for SOT recipients [24,27,36,37,38,39]. Although we only evaluated data during the first six months post-transplant, preemptive multigene panel results would likely have clinical utility over time, given the improved long-term survival of SOT recipients.

To our knowledge, this is the first study to compare PGx medication prescribing patterns and actionable exposure frequencies across different types of SOT. Lung transplant patients exhibited the highest frequency of actionable exposures for all medications, followed by heart and kidney transplant recipients. Moreover, significant differences in PGx medication actionable exposures were observed between transplant types for specific medications. For instance, the frequency of statin actionable exposures was notably higher in kidney transplant recipients compared to lung and heart transplant recipients. This difference is likely due to variations in prescribing patterns and actionable phenotypes for individual statins. Atorvastatin was prescribed to 41.5% of kidney transplant recipients, while pravastatin was prescribed to 100% of heart transplant recipients and 37.1% of lung transplant recipients. Actionable phenotypes for atorvastatin included both SLCO1B1 decreased and poor function, whereas SLCO1B1 poor function was the only actionable phenotype for pravastatin. Similarly, the frequency of actionable exposure to voriconazole was significantly different by transplant type, with heart transplant recipients having the highest actionable exposure, followed by lung and kidney transplant recipients. These findings suggest that it is important to consider the different prescribing patterns and actionable exposure frequencies by transplant type to effectively guide PGx testing and implementation strategies.

## 5. Limitations

There are limitations to our study that deserve acknowledgement. First, our analysis did not include all genes that have been reported in CPIC guidelines, such as *CYP2D6*. Unfortunately, *CYP2D6* was not interrogated on our array in a way that would yield accurate phenotype assignments. Considering that CYP2D6 metabolizes approximately 20% of clinically available medications, the prevalence of actionable exposure to PGx medication in SOT is likely higher than what we have reported in this study [40]. Besides *CYP2D6*, we could not directly genotype or impute a few actionable PGx variants, such as CYP3A5*7 and TPMT *4. The CYP3A5*7 (non-expressor) allele is mainly present in individuals of African ancestry [41], and only 4.7% of our patients were African American; therefore, we may have overestimated the CYP3A5 expressor phenotype in our cohort. TPMT *4 is extremely rare in the general population [12], so the risk of underestimating *TPMT* actionable phenotypes is likely to be low. Another limitation of our study is that it relied on medication prescribing data; therefore, we cannot draw conclusions about medication fills and administration. Finally, our cohort consisted primarily of SOT recipients of European descent; therefore, our findings cannot be generalized to populations with more diverse racial backgrounds.

## 6. Conclusions

We observed a high rate of PGx medication prescribing among SOT patients, with nearly half of the patients having at least one actionable exposure to PGx medications. This finding underscores the potential clinical utility of PGx in SOT recipients. Additional PGx studies are needed to evaluate the impact on clinical outcomes and determine the optimal timing of testing in the SOT patient population.

## Figures and Tables

**Table 1:** Patient demographics and clinical characteristics.

Characteristics	N = 358
Age at transplant, years	52.7 ± 13.2
Men	208 (58.1%)
**Race**	
European descent	291 (81.3%)
African American	17 (4.7%)
Asian	8 (2.2%)
American Indian and Alaska native	3 (0.8%)
Other Pacific Islander	2 (0.6%)
Multiple Races	17 (4.7%)
Other	19 (5.3%)
Not reported	1 (0.3%)
**Ethnicity**	
Hispanic	58 (16.2%)
**Transplanted Organ**	
Kidney	258 (72.1%)
Lung	62 (17.3%)
Heart	38 (10.6%)

**Table 2:** Phenotype and diplotype frequencies of the 13 evaluated pharmacogenetic genes in solid organ transplant recipients in the Colorado Center for Personalized Medicine biobank (N = 358).

Gene	Phenotype	Genotype/Diplotype/Activity Score	Frequency
*ABCG2* *rs2231142*	Normal function Intermediate function Poor function	GG GT TT	77.7% 21.5% 0.8%
CYP2B6 *	Ultrarapid metabolizer Rapid metabolizer Normal metabolizer Intermediate metabolizer Poor metabolizer	*4/*4, *4/*22, *22/*22 *1/*4, *1*22 *1/*1 *1/*6, *1/*9, *1/*18, *4/*6, *6/*22 *6/*6, *6/*9	0% 3.9% 48.9% 41.1% 6.1%
CYP2C9 *	Normal metabolizer Intermediate metabolizer Poor metabolizer	*1/*1 *1/*2, *1/*3, *1/*8, *1/*11, *2/*2 *2/*3, *3/*3	65.4% 32.7% 2.0%
CYP2C19 *	Ultrarapid metabolizer Rapid metabolizer Normal metabolizer Intermediate metabolizer Poor metabolizer	*17/*17 *1/*17 *1/*1 *1/*2, *2/*17 *2/*2, *2/*3	2.8% 26.0% 45.8% 23.7% 1.7%
*CYP2C* cluster *rs12777823*	Normal activity Decreased activity Poor activity	GG GA AA	73.5% 25.7% 0.8%
CYP3A5 *	Normal metabolizer (expressor) Intermediate metabolizer (expressor) Poor metabolizer (non-expressor)	*1/*1 *1/*3, *1/*6 *3/*3, *3/*6, *6/*6	2.0% 15.6% 82.4%
*CYP4F2* *rs2108622*	Normal function Intermediate function Poor function	*1/*1 *1/*3 *3/*3	53.9% 40.2% 5.9%
DPYD *	Normal metabolizer Intermediate metabolizer Poor metabolizer	Activity score: 2 Activity score: 1–1.5 Activity score: 0–0.5	95.5% 4.5% 0%
*SLCO1B1* *rs4149056*	Normal function Decreased function Poor function	*1/*1 *1/*5 *5/*5	69.0% 27.9% 3.1%
NUDT15 *	Normal metabolizer Intermediate metabolizer Poor metabolizer	*1/*1 *1/*3 *3/*3	96.1% 3.9% 0%
TPMT *	Normal metabolizer Intermediate metabolizer Poor metabolizer	*1/*1 *1/*2, *1/*3A, *1/*3C *3A/*3A	90.8% 8.9% 0.3%
*UGT1A1* *rs887829*	Normal metabolizer Intermediate metabolizer Poor metabolizer	*1/*1 *1/*80 *80/*80	46.9% 41.3% 11.7%
*VKORC1* *rs9923231*	Low warfarin sensitivity Intermediate warfarin sensitivity Warfarin sensitive	GG GA AA	43.0% 44.7% 12.3%

* Variants interrogated for genes containing haplotypes: *CYP2B6*, rs28399499, rs34223104, rs2279343, rs3745274; *CYP2C19*, rs12248560, rs4244285, rs4986893; *CYP2C9*, rs1799853, rs1057910, rs7900194, rs28371685, rs28371686; *CYP3A5*, rs776746, rs10264272; *DPYD*, rs67376798, rs3918290, rs55886062, rs75017182; *NUDT15*, rs116855232, rs147390019; and *TPMT*, rs1142345, rs1800460, rs1800462.

**Table 3:** Pharmacogenetic medication prescribing and actionable exposure frequencies in solid organ transplant recipients in the Colorado Center for Personalized Medicine biobank during the first six months post-transplant (N = 358).

Drug Class or Drug	Number (%) of Patients with at Least One Prescription During the First Six Months Post-Transplant	Number (%) of Patients with at Least One Actionable Exposure to the Medication During the First Six Months Post-Transplant	Actionable Phenotypes and Number (%) of Patients with an Actionable Exposure
Any PPI use	342 (95.5%)	54 (15.8%)	• CYP2C19 ultrarapid metabolizer, N = 10 (18.5%) • CYP2C19 rapid metabolizer and H. pylori infection or erosive esophagitis, N = 3 (5.6%) • CYP2C19 normal metabolizer and H. pylori infection or erosive esophagitis, N = 1 (1.9%) • CYP2C19 intermediate metabolizer and long-term PPI use, N = 37 (68.5%) • CYP2C19 poor metabolizer and long-term PPI use, N = 3 (5.6%)
• Pantoprazole	260 (72.6%)	43 (16.5%)	• CYP2C19 ultrarapid metabolizer, N = 7 (16.3%) • CYP2C19 rapid metabolizer and H. pylori infection, N = 1 (2.3%) • CYP2C19 normal metabolizer and H. pylori infection, N = 1 (2.3%) • CYP2C19 intermediate metabolizer and long-term pantoprazole use, N = 31 (72%) • CYP2C19 poor metabolizer and long-term pantoprazole use, N = 3 (7%)
• Omeprazole	86 (24.0%)	11 (12.8%)	• CYP2C19 ultrarapid metabolizer, N = 3 (27.3%) • CYP2C19 rapid metabolizer and H. pylori infection or erosive esophagitis, N = 2 (18.2%) • CYP2C19 intermediate metabolizer and long-term omeprazole use, N = 6 (54.5%)
• Lansoprazole	8 (2.2%)	2 (25%)	• CYP2C19 intermediate metabolizer and long-term lansoprazole use, N = 2 (100%)
Tacrolimus	310 (86.6%)	55 (17.7%)	• CYP3A5 normal metabolizer, N = 7 (12.7%) • CYP3A5 intermediate metabolizer, N = 48 (87.3%)
Any statin use	214 (59.8%)	46 (21.5%)	• SLCO1B1 decreased function, N = 38 (82.6%) • SLCO1B1 poor function, N = 7 (15.2%) • SLCO1B1 decreased function and ABCG2 poor function, N = 1 (2.2%)
• Atorvastatin	128 (35.8%)	36 (28.1%)	• SLCO1B1 decreased function, n = 33 (91.7%) • SLCO1B1 poor function, N = 3 (8.3%)
• Pravastatin	79 (22.1%)	1 (1.3%)	• SLCO1B1 Poor function, N = 1 (100%)
• Simvastatin	26 (7.3%)	7 (26.9%)	• SLCO1B1 decreased function, N = 5 (71.4%) • SLCO1B1 poor function, N = 2 (28.6%)
• Rosuvastatin	12 (3.4%)	2 (16.7%)	• SLCO1B1 decreased function and ABCG2 poor function, N = 1 (50%) • SLCO1B1 poor function and ABCG2 normal function, N = 1 (50%)
Azathioprine	65 (18.2%)	9 (13.8%)	• TPMT intermediate metabolizer and NUDT15 normal metabolizer, N = 6 (66.7%) • TPMT normal metabolizer and NUDT15 intermediate metabolizer, N = 3 (33.3%)
Warfarin	47 (13.1%)	42 (89.4%)	• VKORC1 altered warfarin sensitivity and CYP2C9 normal metabolizer, N = 11 (26.2%) • VKORC1 altered warfarin sensitivity and CYP2C9 normal metabolizer and CYP4F2 non-normal function, N = 7 (16.7%) • VKORC1 altered warfarin sensitivity and CYP2C9 non-normal metabolizer, N = 4 (9.5%) • VKORC1 altered warfarin sensitivity and CYP2C9 non-normal metabolizer CYP4F2 non-normal function, N = 5 (11.9%) • VKORC1 normal warfarin sensitivity and CYP2C9 non-normal metabolizer, N = 5 (11.9%) • VKORC1 normal warfarin sensitivity and CYP2C9 non-normal metabolizer and CYP4F2 non-normal function, N = 5 (11.9%) • VKORC1 normal warfarin sensitivity and CYP2C9 normal metabolizer and CYP4F2 non-normal function, N = 5 (11.9%)
Any SSRI use	41 (11.5%)	22 (53.7%)	• CYP2C19 ultrarapid metabolizer, N = 1 (4.5%) • CYP2C19 rapid metabolizer, N = 8 (36.4%) • CYP2C19 intermediate metabolizer, N = 9 (40.9%) • CYP2C19 intermediate metabolizer and CYP2B6 intermediate metabolizer, N = 1 (4.5%) • CYP2C19 intermediate metabolizer and CYP2B6 poor metabolizer, N = 1 (4.5%) • CYP2C19 poor metabolizer and CYP2B6 normal metabolizer, N = 2 (9.1%)
• Citalopram	22 (6.1%)	14 (63.6%)	• CYP2C19 ultrarapid metabolizer, N = 1 (7.1%) • CYP2C19 rapid metabolizer, N = 8 (57.1%) • CYP2C19 intermediate metabolizer, N = 5 (35.7%)
• Escitalopram	9 (2.5%)	6 (66.7%)	• CYP2C19 rapid metabolizer, N = 3 (50%) • CYP2C19 intermediate metabolizer, N = 3 (50%)
• Sertraline	19 (5.3%)	8 (42.1%)	• CYP2C19 intermediate metabolizer and CYP2B6 normal metabolizer, N = 4 (50%) • CYP2C19 intermediate metabolizer and CYP2B6 intermediate metabolizer, N = 1 (12.5%) • CYP2C19 intermediate metabolizer and CYP2B6 poor metabolizer, N = 1 (12.5%) • CYP2C19 poor metabolizer and CYP2B6 normal metabolizer, N = 2 (25%)
Voriconazole	17 (4.7%)	8 (47.1%)	• CYP2C19 ultrarapid metabolizer, N = 1 (12.5%) • CYP2C19 rapid metabolizer, N = 7 (87.5%)
Clopidogrel	5 (1.4%)	1 (20%)	• CYP2C19 intermediate metabolizer; N = 1 (100%)
Hydantoins	1 (0.3%)	1 (100%)	• CYP2C9 intermediate metabolizer, AS = 1; N = 1 (100%)
• Phenytoin	1 (0.3%)	1 (100%)	• CYP2C9 intermediate metabolizer, AS = 1; n = 1 (100%)
• Fosphenytoin	1 (0.3%)	1 (100%)	• CYP2C9 intermediate metabolizer, AS = 1; n = 1 (100%)

PPI, proton pump inhibitor; SSRI, selective serotonin reuptake inhibitor.

## Data Availability

The datasets used in this study contain protected health information and are not readily available to the public. Requests to access de-identified data should be directed to the corresponding author.

## Supplementary Materials

The following are available online at https://www.mdpi.com/article/10.3390/jpm15050185/s1, Table S1: Drug-gene pairs evaluated in this study (N = 42), Table S2: Comparison of PGx medication prescribing and actionable exposure frequencies in the first six months post-transplant among kidney (N = 258), heart (N = 38), and lung (N = 62) transplant recipients.

## Abbreviations

The following abbreviations are used in this manuscript:

CCPM	Colorado Center for Personalized Medicine
CPIC	Clinical Pharmacogenetics Implementation Consortium
HDC	Health Data Compass
PGx	Pharmacogenetics
PPIs	Proton Pump Inhibitors
SOT	Solid Organ Transplantation
SSRIs	Selective Serotonin reuptake Inhibitors

## References

[1] Black C.K., Termanini K.M., Aguirre O., Hawksworth J.S., Sosin M. (2018). Solid organ transplantation in the 21(st) century. Ann. Transl. Med..

[2] Schladt D.P., Israni A.K. (2025). OPTN/SRTR 2023 Annual Data Report: Introduction. Am. J. Transplant..

[3] Holt C.D. (2017). Overview of Immunosuppressive Therapy in Solid Organ Transplantation. Anesthesiol. Clin..

[4] Graham C.N., Watson C., Barlev A., Stevenson M., Dharnidharka V.R. (2022). Mean lifetime survival estimates following solid organ transplantation in the US and UK. J. Med. Econ..

[5] Singh T.P., Cherikh W.S., Hsich E., Lewis A., Perch M., Kian S., Hayes D., Potena L., Stehlik J., Zuckermann A. (2023). Graft survival in primary thoracic organ transplant recipients: A special report from the International Thoracic Organ Transplant Registry of the International Society for Heart and Lung Transplantation. J. Heart Lung Transplant..

[6] Gomis-Pastor M., Roig Mingell E., Mirabet Perez S., Brossa Loidi V., Lopez Lopez L., Diaz Bassons A., Aretio Pousa A., Feliu Ribera A., Ferrero-Gregori A., Guirado Perich L. (2019). Multimorbidity and medication complexity: New challenges in heart transplantation. Clin. Transplant..

[7] Bryant B.M., Libby A.M., Metz K.R., Page R.L., Ambardekar A.V., Lindenfeld J., Aquilante C.L. (2016). Evaluating Patient-Level Medication Regimen Complexity Over Time in Heart Transplant Recipients. Ann. Pharmacother..

[8] Marienne J., Laville S.M., Caillard P., Batteux B., Gras-Champel V., Masmoudi K., Choukroun G., Liabeuf S. (2021). Evaluation of Changes Over Time in the Drug Burden and Medication Regimen Complexity in ESRD Patients Before and After Renal Transplantation. Kidney Int. Rep..

[9] Roden D.M., McLeod H.L., Relling M.V., Williams M.S., Mensah G.A., Peterson J.F., Van Driest S.L. (2019). Pharmacogenomics. Lancet.

[10] Relling M.V., Klein T.E. (2011). CPIC: Clinical Pharmacogenetics Implementation Consortium of the Pharmacogenomics Research Network. Clin. Pharmacol. Ther..

[11] Birdwell K.A., Decker B., Barbarino J.M., Peterson J.F., Stein C.M., Sadee W., Wang D., Vinks A.A., He Y., Swen J.J. (2015). Clinical Pharmacogenetics Implementation Consortium (CPIC) Guidelines for CYP3A5 Genotype and Tacrolimus Dosing. Clin. Pharmacol. Ther..

[12] Relling M.V., Schwab M., Whirl-Carrillo M., Suarez-Kurtz G., Pui C.H., Stein C.M., Moyer A.M., Evans W.E., Klein T.E., Antillon-Klussmann F.G. (2019). Clinical Pharmacogenetics Implementation Consortium Guideline for Thiopurine Dosing Based on TPMT and NUDT15 Genotypes: 2018 Update. Clin. Pharmacol. Ther..

[13] Cooper-DeHoff R.M., Niemi M., Ramsey L.B., Luzum J.A., Tarkiainen E.K., Straka R.J., Gong L., Tuteja S., Wilke R.A., Wadelius M. (2022). The Clinical Pharmacogenetics Implementation Consortium Guideline for SLCO1B1, ABCG2, and CYP2C9 genotypes and Statin-Associated Musculoskeletal Symptoms. Clin. Pharmacol. Ther..

[14] Lima J.J., Thomas C.D., Barbarino J., Desta Z., Van Driest S.L., El Rouby N., Johnson J.A., Cavallari L.H., Shakhnovich V., Thacker D.L. (2021). Clinical Pharmacogenetics Implementation Consortium (CPIC) Guideline for CYP2C19 and Proton Pump Inhibitor Dosing. Clin. Pharmacol. Ther..

[15] Bell G.C., Caudle K.E., Whirl-Carrillo M., Gordon R.J., Hikino K., Prows C.A., Gaedigk A., Agundez J., Sadhasivam S., Klein T.E. (2017). Clinical Pharmacogenetics Implementation Consortium (CPIC) guideline for CYP2D6 genotype and use of ondansetron and tropisetron. Clin. Pharmacol. Ther..

[16] Johnson J.A., Caudle K.E., Gong L., Whirl-Carrillo M., Stein C.M., Scott S.A., Lee M.T., Gage B.F., Kimmel S.E., Perera M.A. (2017). Clinical Pharmacogenetics Implementation Consortium (CPIC) Guideline for Pharmacogenetics-Guided Warfarin Dosing: 2017 Update. Clin. Pharmacol. Ther..

[17] Lee C.R., Luzum J.A., Sangkuhl K., Gammal R.S., Sabatine M.S., Stein C.M., Kisor D.F., Limdi N.A., Lee Y.M., Scott S.A. (2022). Clinical Pharmacogenetics Implementation Consortium Guideline for CYP2C19 Genotype and Clopidogrel Therapy: 2022 Update. Clin. Pharmacol. Ther..

[18] Moriyama B., Obeng A.O., Barbarino J., Penzak S.R., Henning S.A., Scott S.A., Agúndez J., Wingard J.R., McLeod H.L., Klein T.E. (2017). Clinical Pharmacogenetics Implementation Consortium (CPIC) Guidelines for CYP2C19 and Voriconazole Therapy. Clin. Pharmacol. Ther..

[19] Crews K.R., Monte A.A., Huddart R., Caudle K.E., Kharasch E.D., Gaedigk A., Dunnenberger H.M., Leeder J.S., Callaghan J.T., Samer C.F. (2021). Clinical Pharmacogenetics Implementation Consortium Guideline for CYP2D6, OPRM1, and COMT Genotypes and Select Opioid Therapy. Clin. Pharmacol. Ther..

[20] Bousman C.A., Stevenson J.M., Ramsey L.B., Sangkuhl K., Hicks J.K., Strawn J.R., Singh A.B., Ruaño G., Mueller D.J., Tsermpini E.E. (2023). Clinical Pharmacogenetics Implementation Consortium (CPIC) Guideline for CYP2D6, CYP2C19, CYP2B6, SLC6A4, and HTR2A Genotypes and Serotonin Reuptake Inhibitor Antidepressants. Clin. Pharmacol. Ther..

[21] Hicks J.K., Sangkuhl K., Swen J.J., Ellingrod V.L., Müller D.J., Shimoda K., Bishop J.R., Kharasch E.D., Skaar T.C., Gaedigk A. (2017). Clinical pharmacogenetics implementation consortium guideline (CPIC) for CYP2D6 and CYP2C19 genotypes and dosing of tricyclic antidepressants: 2016 update. Clin. Pharmacol. Ther..

[22] McInnes G., Lavertu A., Sangkuhl K., Klein T.E., Whirl-Carrillo M., Altman R.B. (2021). Pharmacogenetics at Scale: An Analysis of the UK Biobank. Clin. Pharmacol. Ther..

[23] Nguyen T.T., Pearson R.A., Mohamed M.E., Schladt D.P., Berglund D., Rivers Z., Skaar D.J., Wu B., Guan W., van Setten J. (2020). Pharmacogenomics in kidney transplant recipients and potential for integration into practice. J. Clin. Pharm. Ther..

[24] Wang L., Scherer S.E., Bielinski S.J., Muzny D.M., Jones L.A., Black J.L., Moyer A.M., Giri J., Sharp R.R., Matey E.T. (2022). Implementation of preemptive DNA sequence-based pharmacogenomics testing across a large academic medical center: The Mayo-Baylor RIGHT 10K Study. Genet. Med..

[25] Chang Y.L., Hsiao T.H., Wu M.F., Chen C.H. (2023). The Prevalence and Features of Medications with Actionable Pharmacogenomic Biomarkers Prescribed to Kidney Transplant Recipients. Transplant. Proc..

[26] Brady A., Misra S., Abdelmalek M., Kekic A., Kunze K., Lim E., Jakob N., Mour G., Keddis M.T. (2023). The Value of Pharmacogenomics for White and Indigenous Americans after Kidney Transplantation. Pharmacy.

[27] Aquilante C.L., Kao D.P., Trinkley K.E., Lin C.T., Crooks K.R., Hearst E.C., Hess S.J., Kudron E.L., Lee Y.M., Liko I. (2020). Clinical implementation of pharmacogenomics via a health system-wide research biobank: The University of Colorado experience. Pharmacogenomics.

[28] Wiley L.K., Shortt J.A., Roberts E.R., Lowery J., Kudron E., Lin M., Mayer D., Wilson M., Brunetti T.M., Chavan S. (2024). Building a vertically integrated genomic learning health system: The biobank at the Colorado Center for Personalized Medicine. Am. J. Hum. Genet..

[29] Illumina I.I. Expanded Multi-Ethnic GenotypingArray (MEGAEX). www.illumina.com/content/dam/illumina-marketing/documents/products/datasheets/mega-ex-data-sheet-370-2015-004.pdf.

[30] Oreschak K., Saba L.M., Rafaels N., Ambardekar A.V., Deininger K.M., Page I.R., Lindenfeld J., Aquilante C.L. (2021). Variants in mycophenolate and CMV antiviral drug pharmacokinetic and pharmacodynamic genes and leukopenia in heart transplant recipients. J. Heart Lung Transplant..

[31] Das S., Forer L., Schönherr S., Sidore C., Locke A.E., Kwong A., Vrieze S.I., Chew E.Y., Levy S., McGue M. (2016). Next-generation genotype imputation service and methods. Nat. Genet..

[32] PGx Gene-Specific Information Tables. https://www.pharmgkb.org/page/pgxGeneRef.

[33] Caudle K.E., Klein T.E., Hoffman J.M., Muller D.J., Whirl-Carrillo M., Gong L., McDonagh E.M., Sangkuhl K., Thorn C.F., Schwab M. (2014). Incorporation of pharmacogenomics into routine clinical practice: The Clinical Pharmacogenetics Implementation Consortium (CPIC) guideline development process. Curr. Drug Metab..

[34] van Gelder T., Gelinck A., Meziyerh S., de Vries A.P.J., Moes D. (2024). Therapeutic drug monitoring of tacrolimus after kidney transplantation: Trough concentration or area under curve-based monitoring?. Br. J. Clin. Pharmacol..

[35] Brunet M., van Gelder T., Åsberg A., Haufroid V., Hesselink D.A., Langman L., Lemaitre F., Marquet P., Seger C., Shipkova M. (2019). Therapeutic Drug Monitoring of Tacrolimus-Personalized Therapy: Second Consensus Report. Ther. Drug Monit..

[36] Jackson R.L., Heyrend C., Bucher B., Brewer A., Peterson C., May L.J., Bonkowsky J.L. (2025). Impact of Pharmacogenomic Testing in Pediatric Heart and Kidney Transplant. Pediatr. Transplant..

[37] Tamraz B., Shin J., Khanna R., Van Ziffle J., Knowles S., Stregowski S., Wan E., Kamath R., Collins C., Phunsur C. (2025). Clinical implementation of preemptive pharmacogenomics testing for personalized medicine at an academic medical center. J. Am. Med. Inform. Assoc..

[38] Liu M., Vnencak-Jones C.L., Roland B.P., Gatto C.L., Mathe J.L., Just S.L., Peterson J.F., Van Driest S.L., Weitkamp A.O. (2021). A Tutorial for Pharmacogenomics Implementation Through End-to-End Clinical Decision Support Based on Ten Years of Experience from PREDICT. Clin. Pharmacol. Ther..

[39] Shugg T., Tillman E.M., Breman A.M., Hodge J.C., McDonald C.A., Ly R.C., Rowe E.J., Osei W., Smith T.B., Schwartz P.H. (2024). Development of a Multifaceted Program for Pharmacogenetics Adoption at an Academic Medical Center: Practical Considerations and Lessons Learned. Clin. Pharmacol. Ther..

[40] Taylor C., Crosby I., Yip V., Maguire P., Pirmohamed M., Turner R.M. (2020). A Review of the Important Role of CYP2D6 in Pharmacogenomics. Genes.

[41] Lamba J., Hebert J.M., Schuetz E.G., Klein T.E., Altman R.B. (2012). PharmGKB summary: Very important pharmacogene information for CYP3A5. Pharmacogenetics Genom..

