# Supplementary material for: Prevalence of Actionable Exposures to Pharmacogenetic Medications Among Solid Organ Transplant Recipients in a Population-Scale Biobank
Source: J Pers Med. 2025 May 2;15(5):185. doi: 10.3390/jpm15050185 (PMC12113073; doi:10.3390/jpm15050185)
Supplement: Supplementary file 1 [file jpm-15-00185-s001.zip › jpm-3580007-supplementary.pdf]

Supplementary Table S1: Drug-gene pairs evaluated in this study (N=42)

Medication	Genes
Amitriptyline	*CYP2C19*
Atazanavir	*UGT1A1*
Atorvastatin	*SLCO1B1*
Azathioprine	*TPMT, NUDT15*
Belinostat	*UGT1A1*
Brivaracetam	*CYP2C19*
Capecitabine	*DPYD*
Celecoxib	*CYP2C9*
Citalopram	*CYP2C19*
Clomipramine	*CYP2C19*
Clopidogrel	*CYP2C19*
Dexlansoprazole	*CYP2C19*
Doxepin	*CYP2C19*
Efavirenz	*CYP2B6*
Escitalopram	*CYP2C19*
Fluorouracil	*DPYD*
Flurbiprofen	*CYP2C9*
Fluvastatin	*SLCO1B1*
Fosphenytoin	*CYP2C9*
Ibuprofen	*CYP2C9*
Imipramine	*CYP2C19*
Irinotecan	*UGT1A1*
Lansoprazole	*CYP2C19*
Lovastatin	*SLCO1B1*
Meloxicam	*CYP2C9*
Mercaptopurine	*TPMT, NUDT15*
Omeprazole	*CYP2C19*
Pantoprazole	*CYP2C19*
Phenytoin	*CYP2C9*
Piroxicam	*CYP2C9*
Pitavastatin	*SLCO1B1*
Pravastatin	*SLCO1B1*
Rosuvastatin	*SLCO1B1, ABCG2*
Sertraline	*CYP2C19, CYP2B6*
Simvastatin	*SLCO1B1*
Siponimod	*CYP2C9*
Tacrolimus	*CYP3A5*
Tenoxicam	*CYP2C9*
Thioguanine	*TPMT, NUDT15*
Trimipramine	*CYP2C19*
Voriconazole	*CYP2C19*
Warfarin	*VKORC1, CYP2C9, CYP4F2, rs12777823*

Supplementary Table S2: Comparison of PGx medication prescribing and actionable exposure frequencies in the first six months post-transplant among kidney (N=258), heart (N=38), and lung (N=62) transplant recipients

**Drug Class or Drug**	**Number (%) of patients with at least one prescription**				**Number (%) of patients with at least one actionable exposure to the medication***			
	**Kidney N=258**	**Heart N=38**	**Lung N=62**	**P-value**	**Kidney N=258**	**Heart N=38**	**Lung N=62**	**P-value**
Any PPI use	245 (95.0%)	37 (97.4%)	60 (96.8%)	0.92	34 (13.9%)	8 (21.6%)	12 (20%)	0.30
• Pantoprazole	190 (73.6%)	30 (78.9%)	40 (64.5%)	0.23	26 (13.7%)	7 (23.3%)	10 (25%)	0.12
• Omeprazole	58 (22.5%)	7 (18.4%)	21 (33.9%)	0.12	8 (13.8%)	2 (28.6%)	1 (4.8%)	1
• Lansoprazole	0	2 (5.3%)	6 (9.7%)	<0.001	0	1 (50%)	1 (16.7%)	0.46
Tacrolimus	241 (93.4%)	35 (92.1%)	34 (54.8%)	<0.001	41 (17.0%)	9 (25.7%)	5 (14.7%)	0.40
Any statin use	147 (57%)	38 (100%)	29 (46.8%)	<0.001	40 (27.2%)	2 (5.3%)	4 (13.8%)	0.005
• Atorvastatin	107 (41.5%)	10 (26.3%)	11 (17.7%)	<0.001	31 (29%)	2 (20%)	3 (27.3%)	0.92
• Pravastatin	18 (7%)	38 (100%)	23 (37.1%)	<0.001	0	0	1 (4.3%)	0.52
• Simvastatin	25 (9.7%)	0	1 (1.6%)	0.012	7 (28%)	0	0	1
• Rosuvastatin	10 (3.9%)	1 (2.6%)	1 (1.6%)	0.89	2 (20%)	0	0	1
Azathioprine	11 (4.3%)	0	54 (78.1%)	<0.001	2 (18.2%)	0	7 (13%)	0.64
Warfarin	28 (10.9%)	5 (13.2%)	14 (22.6%)	0.049	23 (82.1%)	5 (100%)	14 (100%)	0.20
Any SSRI use	23 (8.9%)	6 (15.8%)	12 (19.4%)	0.046	11 (47.8%)	5 (83.3%)	6 (50%)	0.34
• Citalopram	14 (5.4%)	2 (5.3%)	6 (9.7%)	0.44	7 (50%)	2 (100%)	5 (83.3%)	0.31
• Escitalopram	7 (2.7%)	0	2 (3.2%)	0.64	4 (57.1%)	0	2 (100%)	0.5
• Sertraline	9 (3.5%)	4 (10.5%)	6 (9.7%)	0.047	4 (44.4%)	3 (75%)	1 (16.7%)	0.25
Voriconazole	3 (1.2%)	3 (7.9%)	11 (17.7%)	<0.001	0	3 (100%)	5 (45.5%)	0.047

*Denominators used to calculate the frequencies of actionable exposure to each medication were the number of patients within each organ type with at least one prescription for the medication.

P-values were generated using chi-square tests or Fisher's exact tests. A p-value < 0.05 was considered significant.
